# Supplementary material for: Evaluating Neuroprotective Effects of Uridine, Erythropoietin, and Therapeutic Hypothermia in a Ferret Model of Inflammation-Sensitized Hypoxic-Ischemic Encephalopathy
Source: Int J Mol Sci. 2021 Sep 11;22(18):9841. doi: 10.3390/ijms22189841 (PMC8469346; doi:10.3390/ijms22189841)
Supplement: Supplementary file 1 [file ijms-22-09841-s001.zip › ijms-1332613-SI.pdf]

Supplemental Information - Evaluating Neuroprotective Effects of Uridine, Erythropoietin, and Therapeutic Hypothermia in a Ferret Model of Inflammation-Sensitized Hypoxic-Ischemic Encephalopathy

Supplemental Methods

Plasma Uracil and Uridine Assays

Uracil and Ur were measured using high pressure liquid chromatography-mass spectrometry. The assay was performed on a Water's Xevo-TQs coupled to a Water's I-Class Ultra high-pressure liquid chromatography system (Waters Corporation, Milford, MA, USA). Analytes were monitored in MRM mode acquired using negative electrospray ionization. Chromatographic separation was achieved using a Water's Acquity UPLC BEH Phenyl column, 2.1 × 100 mm, 1.7μ column (Waters Corporation, Milford, MA, USA). Using a gradient consisting of 0.1% acetic acid in H_2O (A) and 0.1% acetic acid in acetonitrile (ACN) (B) at a flow rate 0.3 mL/min with a total run time of 8.5 min. Retention time using the column and gradient was achieved at 1.29 min for Uracil/C13 Uracil and 1.99 min for Ur/C13 Ur. Calibration curves were prepared by spiking stocks of the diluted analytes into water (31.25–1000 ng/mL) due to high levels of endogenous interfering compounds found in plasma. 10 ul of calibrator, QC (100 ng/mL), or unknown plasma sample was placed in an Eppendorf and 10 ul of internal standard mix (1 ug/mL in H_2O) was added to each sample. 30 μL of H_2O was added, vortexed then 500 μL of ethyl acetate:2-propanol (10:1). Sample was vigorously shaken for 5 min then centrifuged at 16 rfm for 5 min. Supernatant was removed and evaporated to dryness under a steady stream on nitrogen. Samples were reconstituted with 50 μL of 0.1% acetic acid in water. Analysis of the data was done using QuanLynxs software (Waters Corporation, Milford, MA, USA) by generating a linear equation based on peak area ratios (PAR) of the *m/z* parent analyte over the internal standard m/z area. This was then compiled against the expected concentration of the calibrators to generate a slope and intercept to be applied back to the PAR's to obtain a return value. Acceptability of levels of quantitation was determined by a variance less than 15% from the expected return value.

MRI

The magnet is fitted with custom [Resonance Research, Billerica, MA], 9 cm i.d., high performance gradients achieving an average of 750 mT/meter gradient with a 100 μsec slew rate. A TurboRARE 3D T2 sequence provided anatomical images using the following settings: Field of view (FOV) 30 mm, slice thickness 0.23 mm, sagittal slice orientation matrix with 1.6 sec recycle time and 39 msec echo time accomplished via a rare factor of 16 and acquired into a 128 point per axis cubic volume. Diffusion tensor images were obtained using a 2D collection with the same FOV as the T2. Slice thickness was 0.6 mm, for 30 diffusion directions [plus 5 A0 images], and eight averages for 64 slices [128 × 128] each 0.6 mm thickness. The recycle time was set to 5.6 sec with an echo time of 48 msec. Diffusion weighting was set to 4 msec gradient duration [δ] and 10 msec gradient separation [Δ] with a maximum B value of 4320 to result in 30 diffusion directions. Diffusion-weighted images were motion- and distortion-corrected using the latest version of FMRIB Software Library (FSL v5.0, Oxford, UK) eddy software (https://fsl.fmrib.ox.ac.uk/fsl/fslwiki/eddy). FSL's dtifit was then applied, and the resulting diffusion tensor image (DTI) median filtered using fslmaths-fmedian option. The resulting output files were fractional anisotropy (FA), L1, L2, L3 (the three eigenvalue maps), MD (mean diffusivity), and MO (dti mode) maps. Radial diffusivity was calculated by combining the L2 and L3 maps. Co-

registration of the FA maps from all subjects was performed using tract-based spatial statistics (TBSS). Comparisons of TBSS between groups were Threshold-Free Cluster Enhancement (TFCE) adjusted for multiple comparisons.

Exploration Index

An exploration index was developed to expand upon the automated water maze outcomes. Image files of each ferret's complete swim path were extracted from the EthoVisionXT software. The area of the ferret's swim path was measured using ImageJ and adjusted for the total distance traveled (cm) and the total swim time (s) in an attempt to quantify the ferret's extent of swimming tub exploration.

Supplemental Figures

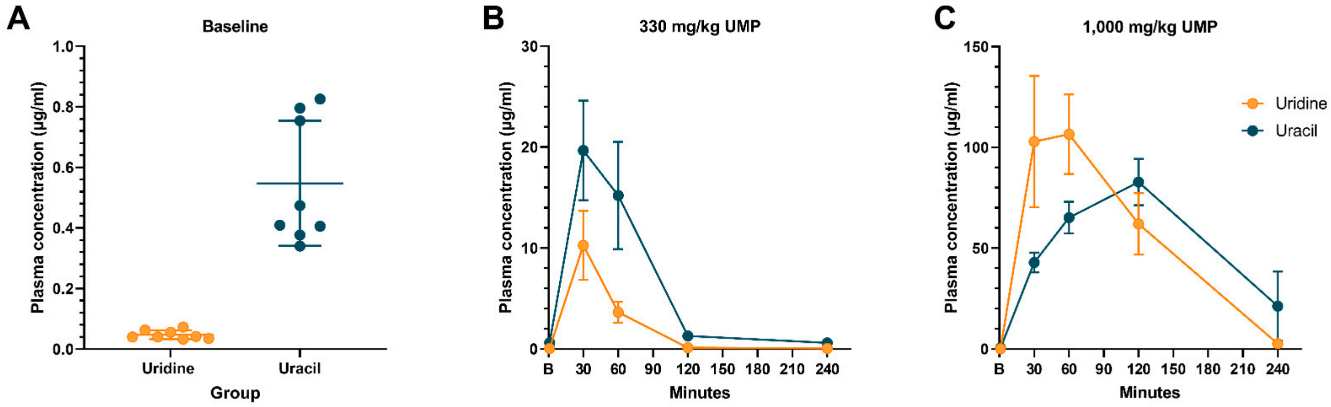

Supplemental Figure S1. Uridine and uracil plasma concentrations. (**A**) Scatter plot of baseline uridine and uracil plasma concentrations in micrograms per milliliter (µg/mL). The baseline levels of uracil were 10× higher than the levels of uridine. (**B**) Plot of the mean and standard deviation for uridine and uracil plasma concentrations. Uridine plasma concentrations peak 30 min after injecting 330 mg/kg UMP and return to baseline levels after 120 min. (**C**) Plot of the mean and standard deviation for uridine and uracil plasma concentrations. After receiving a 1000 mg/kg dose of UMP, uridine levels peak at 60 min while uracil levels peak at 120 min.

		Treatment Assignments (survived HIH)		Deaths post-treatment	P42 Survival	
Cohort	Trx	♂	♀	Total	♂	♀
Uridine: 71 experimental animals	Control	10	9	0	10	9
	Vehicle	9	8	3	7	7
	Uridine	11	12	8	8	7
Epo & TH: 115 experimental animals	Control	12	12	0	12	12
	Vehicle	12	13	2	10	13
	Epo	13	13	2	12	12
	TH	12	12	3	10	11

Supplemental Figure S2. Experimental group totals by sex and cohort pre- and post-treatment assignments.

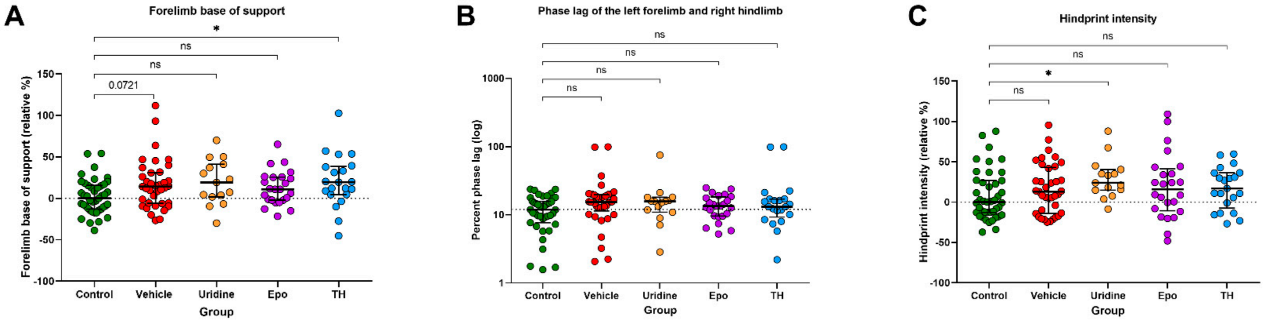

Supplemental Figure S3. CatWalk gait analysis. Scatter plots of six CatWalk metrics. The median with IQR is plotted on each scatter. (**A**) Forelimb base of support (BOS) in the Veh, Ur, Epo, and TH relative to the control median. Median forelimb BOS was 0.01 cm (cm) per gram (g) bodyweight in the control group. Median forelimb BOS significantly increased in the TH group by 19.6% and non-significantly increased in all other HIH-exposed groups. (**B**) Scatter plot of the log transformation of percent phase lag between left forelimb and right hindlimb. Median logged percent phase lag was 11.9% in the control group and non-significantly increased in the Veh and Ur groups. (**C**) Hindprint intensity in the Veh, Ur, Epo, and TH groups relative to the control median. Median hindprint intensity was 0.06 au/g in the control group. Compared to control median, medians were significantly increased in the Ur group by 23.9% and non-significantly increased in all other HIH-exposed groups. * Denotes significant *p*-value <0.05 and ** denotes *p*-value <0.01.

A

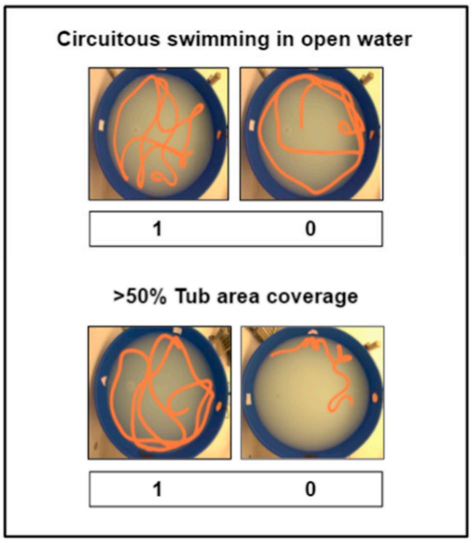

B

Swim Pattern	Rx	Or (95% CI)	p-value
Circuitous swimming in open water	Control	Reference	-
	Veh	0.67 (0.20-2.23)	0.51
	Epo	0.32 (0.10-0.98)	**0.046**
	TH	0.96 (0.27-3.42)	0.95
>50% Tub area coverage	Control	Reference	-
	Veh	0.62 (0.19-2.03)	0.43
	Epo	0.25 (0.07-0.90)	**0.034**
	TH	1.02 (0.32-3.26)	0.98

Supplemental Figure S4. Water maze swim pattern analysis. **(A)** Illustration of qualitative scoring methods for ferret swim patterns during water maze trials. Example images show the water maze tub and orange swim path for that trial. The images were evaluated on a dichotomous scale for circuitous swimming in open water and for >50% tub area coverage. Scores are listed below each image. Swim patterns resembling control-like circuictous swimming in open water or >50% tub area coverage were scored a 1. Swim patterns that did not fall into the two categories were scored a 0. **(B)** Logistic regression analysis for circuitous swimming in open water and for >50% tub area coverage. The Epo group had the lowest likelihood of swimming circuitously in open water and covering >50% of the tub area ($p > 0.05$ for both). Swim patterns of the Veh and TH groups were more likely to resemble control circuitous swimming in open water and >50% tub coverage ($p > 0.05$ for both).

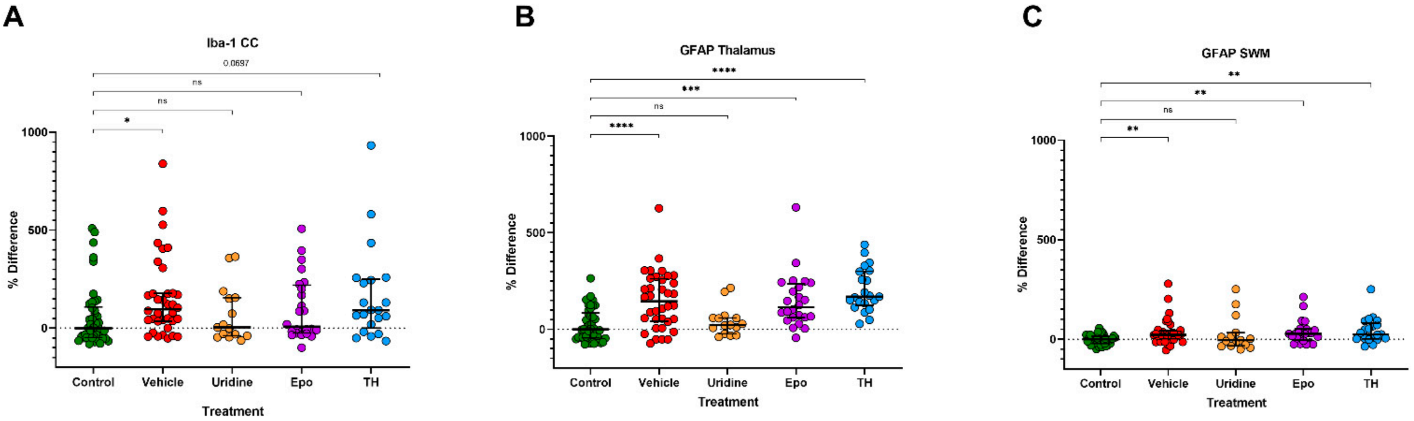

Supplemental Figure S5. Quantitative immunohistochemistry (qIHC) staining. Scatter plots of three qIHC metrics. The median with IQR is plotted on each scatter. (**A**) Corpus collosum (CC) Iba-1 staining in the Veh, Ur, Epo, and TH groups relative to the control group. Within the CC, Iba-1 staining had a median (IQR) increase of 96.3% (35.4–177.6%) in Veh brains relative to the control staining. (**B**) Thalamus GFAP staining in the HIH-exposed groups relative to the control group. In the thalamus, GFAP staining (median; IQR) was increased in the Veh (145%; 42.4–261.7%), Epo (114.1%; 61.9–235.5%), and TH (169%; 124.1–301.3%) brains when compared to controls**.** (**C**) Subcortical white matter GFAP staining in

the HIH groups relative to control median. In the SWM, GFAP staining was increased in the SWM of Veh (21.9%; 2.5–42.7%), Epo (27.2%; –4.4–51.9%), and TH (24.8%; 1.8–81.2%) brains when compared to control brains. *Denotes significant *p*-value <0.05, ** denotes *p*-value <0.01, *** denotes *p*-value <0.001, and **** denotes *p*-value <0.0001.

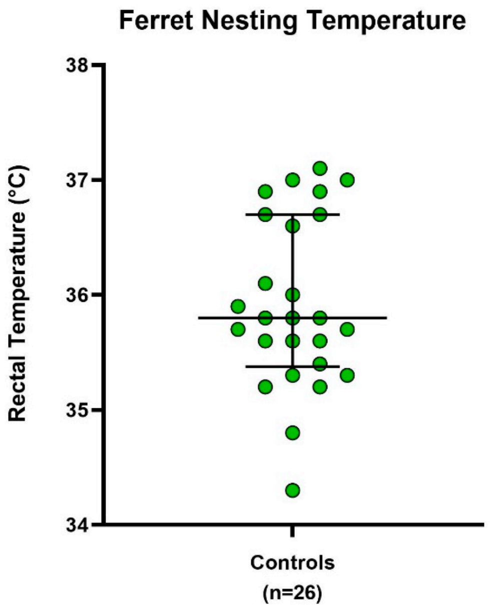

Supplemental Figure S6. Nesting rectal temperatures of control animals at postnatal day 17.

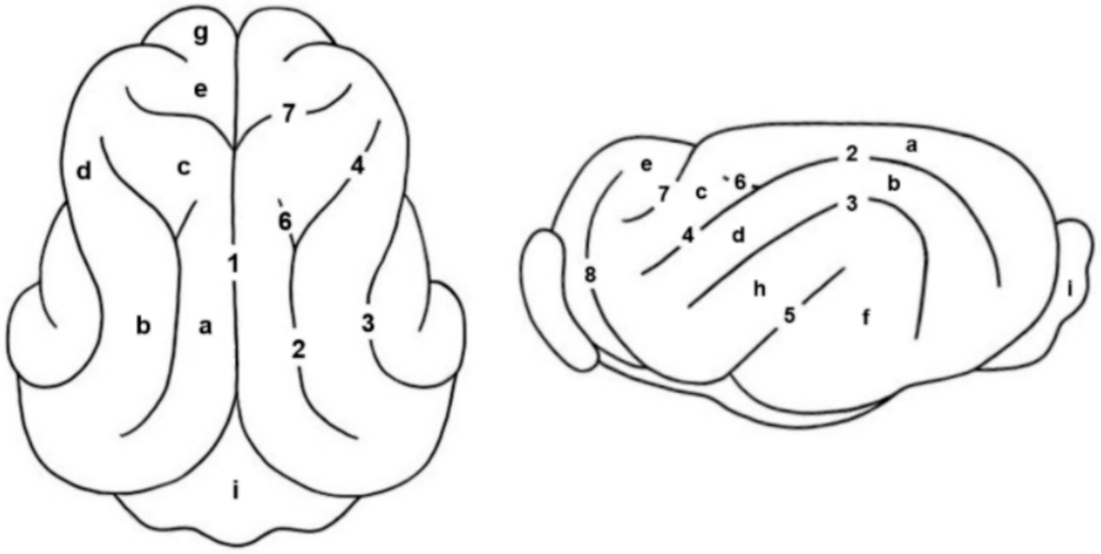

Supplemental Figure S7. Ferret brain atlas**.** The ferret cerebral cortex, containing the following as labeled: 1. Longitudinal Fissure, 2. Lateral Sulcus, 3. Suprasylvian Sulcus, 4. Coronal Sulcus, 5. Pseudosylvian Sulcus, 6. Ansinate Sulcus, 7. Cruciate Sulcus, 8. Presylvian Sulcus, a. Lateral Gyrus, b. Suprasylvian Gyrus, c. Posterior Sigmoid Gyrus, d. Coronal Gyrus, e. Ectosylvian Gyrus, g. Orbital Gyrus, i. Cerebellum exposed.

A B

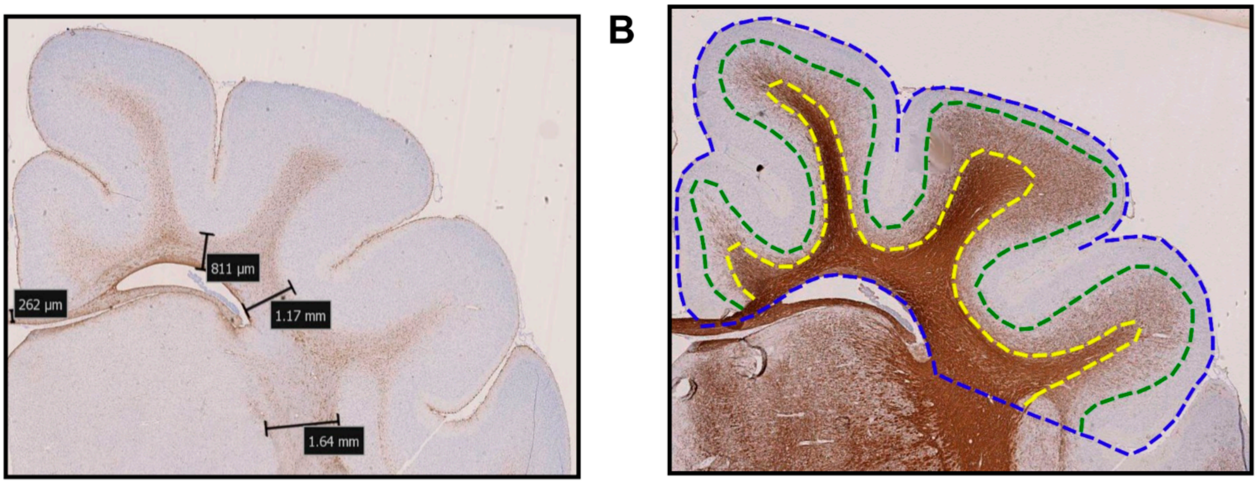

Supplemental Figure S8. White matter tract thickness measurements. (**A**) On GFAP-stained slices, four white matter regions of interest (ROIs) were measured: the corpus callosum (CC) and three white matter regions inferomedial to the lateral sulci, suprasylvian sulci, and pseudosylvian sulci. The CC measurement was taken at the midline of the CC, where the width of the most dorsal and ventral aspects of the CC were measured. The other ROIs were measured from the most inferomedial appearance of white matter from the corresponding sulci to the most medial border of the white matter tracts. Measurements were taken using the NDP.view2 annotation tool. (**B**) On the MBP-stained slices, the areas of three ROIs were measured: the densely myelinated white matter tracts near the innermost white matter structures (yellow), the sparsely myelinated white matter fibers penetrating the gray matter of the outer cortex (green), and the combined area of white and gray matter (blue). Measurements were taken using the ImageJ freehand selection tool was used to trace the ROIs and to obtain the respective areas.
